# New Insights into the Function and Global Distribution of Polyethylene Terephthalate (PET)-Degrading Bacteria and Enzymes in Marine and Terrestrial Metagenomes

**DOI:** 10.1128/AEM.02773-17

**Published:** 2018-04-02

**Authors:** Dominik Danso, Christel Schmeisser, Jennifer Chow, Wolfgang Zimmermann, Ren Wei, Christian Leggewie, Xiangzhen Li, Terry Hazen, Wolfgang R. Streit

**Affiliations:** a Department of Microbiology and Biotechnology, Biocenter Klein Flottbek, University of Hamburg, Hamburg, Germany; b Institute of Biochemistry, Department of Microbiology and Bioprocess Technology, Leipzig University, Leipzig, Germany; c evoxx technologies GmbH, Monheim am Rhein, Germany; d Chengdu Institute of Biology, Chengdu, China; e The University of Tennessee, Knoxville, Tennessee, USA; University of California, Davis

**Keywords:** HMM, hydrolases, metagenome, metagenomic screening, PET degradation, polyethylene terephthalate (PET), BHET, TPA, metagenomes

## Abstract

Polyethylene terephthalate (PET) is one of the most important synthetic polymers used today. Unfortunately, the polymers accumulate in nature and to date no highly active enzymes are known that can degrade it at high velocity. Enzymes involved in PET degradation are mainly α- and β-hydrolases, like cutinases and related enzymes (EC 3.1.1). Currently, only a small number of such enzymes are well characterized. In this work, a search algorithm was developed that identified 504 possible PET hydrolase candidate genes from various databases. A further global search that comprised more than 16 Gb of sequence information within 108 marine and 25 terrestrial metagenomes obtained from the Integrated Microbial Genome (IMG) database detected 349 putative PET hydrolases. Heterologous expression of four such candidate enzymes verified the function of these enzymes and confirmed the usefulness of the developed search algorithm. In this way, two novel and thermostable enzymes with high potential for downstream application were partially characterized. Clustering of 504 novel enzyme candidates based on amino acid similarities indicated that PET hydrolases mainly occur in the phyla of Actinobacteria, Proteobacteria, and Bacteroidetes. Within the Proteobacteria, the Betaproteobacteria, Deltaproteobacteria, and Gammaproteobacteria were the main hosts. Remarkably enough, in the marine environment, bacteria affiliated with the phylum Bacteroidetes appear to be the main hosts of PET hydrolase genes, rather than Actinobacteria or Proteobacteria, as observed for the terrestrial metagenomes. Our data further imply that PET hydrolases are truly rare enzymes. The highest occurrence of 1.5 hits/Mb was observed in sequences from a sample site containing crude oil.

**IMPORTANCE** Polyethylene terephthalate (PET) accumulates in our environment without significant microbial conversion. Although a few PET hydrolases are already known, it is still unknown how frequently they appear and with which main bacterial phyla they are affiliated. In this study, deep sequence mining of protein databases and metagenomes demonstrated that PET hydrolases indeed occur at very low frequencies in the environment. Furthermore, it was possible to link them to phyla that were previously not known to harbor such enzymes. This work contributes novel knowledge on the phylogenetic relationships, the recent evolution, and the global distribution of PET hydrolases. Finally, we describe the biochemical traits of four novel PET hydrolases.

## INTRODUCTION

Since its discovery, its first synthesis, and its patenting in 1941, polyethylene terephthalate (PET) has become a widely used material in several industrial branches (1). The worldwide PET resin production amounted to 27.8 million tons in 2015 (https://www.plasticsinsight.com/global-pet-resin-production-capacity).

Due to its massive use, PET is highly enriched in nature. Microplastics and bigger fragments of plastic are found worldwide in oceans and terrestrial environments. The most prominent example is the so-called Pacific garbage patch. PET debris is often eaten by fish and other marine creatures (2, 3). In this way, PET degradation products and additives (i.e., solubilizers) are introduced into the food chain, where they have negative impact on human and animal health (4). Until now, only a few species of bacteria and fungi have been described as capable of partially degrading PET to oligomers or even monomers (5). Within this framework, however, it is noteworthy that all known PET hydrolases have relatively low turnover rates, which makes their use for efficient bioremediation almost impossible (Table 1).

**TABLE 1:** Currently known and partially characterized PET hydrolases

Sequence no.	PDB entry no.^*a*^	Gene name^*b*^	Organism	Reference
1	W0TJ64	Cut190	Saccharomonospora viridis	6
2	E9LVI0	cut1	Thermobifida fusca (Thermomonospora *fusca*)	7
3	E5BBQ3	cut-2	Thermobifida fusca (Thermomonospora *fusca*)	8
4	D1A9G5	Tcur_1278	Thermomonospora curvata	9
5	E9LVH7	cut1	Thermobifida alba	10
6	H6WX58	NA	Thermobifida halotolerans	11
7	E9LVH9	cut2	Thermobifida celluloysilityca	12
8	A0A0K8P6T7	ISF6_4831	Ideonella sakaiensis	13
9	G9BY57	NA	Uncultured bacterium	14

a Names and protein database (PDB) entry numbers of currently known PET hydrolases used in this work as references. Sequence data of these examples were used for the initial construction of the HMM.

b NA, not applicable.

Intriguingly, the trait for PET degradation appears to be limited to a few bacterial phyla, and most bacterial isolates with potential for PET degradation are members of the Gram-positive phylum Actinobacteria (12). The best-characterized examples originate from the genera Thermobifida and Thermomonospora (8, 10–12, 15, 16) (Table 1). The enzymes involved in the degradation (e.g., PET hydrolase and tannase) are typical serine hydrolases, e.g., cutinases (EC 3.1.1.74), lipases (EC 3.1.1.3), and carboxylesterases (EC 3.1.1.1). These enzymes possess a typical α/β-hydrolase fold, and the catalytic triad is composed of a serine, a histidine, and an aspartate residue (17, 18).

More recently, polyethylene (PE)-degrading bacteria were reported in insect guts. In this recent study, Enterobacteria and Bacillus strains had been isolated and were capable of degrading polyesters (19, 20).

Furthermore, a complete degradation of amorphous PET materials was described for the Gram-negative bacterium Ideonella sakaiensis strain 201-F6, which is able to use PET as a major energy and carbon source (13). In addition to the hydrolase, the I. sakaiensis genome encodes a second enzyme that appears to be unique, and which is designated as a tannase capable of degrading mono(2-hydroxyethyl) terephthalic acid. In this way, the secreted PET hydrolase produces the intermediate mono(2-hydroxyethyl) terephthalic acid (MHET). MHET is presumably internalized by the cell and hydrolyzed by the MHETase. The resulting monomers are then degraded in a downstream process and used for the bacterial metabolism. I. sakaiensis is affiliated with the phylum Betaproteobacteria and belongs to the order Burkholderiales.

**FIG 1:**
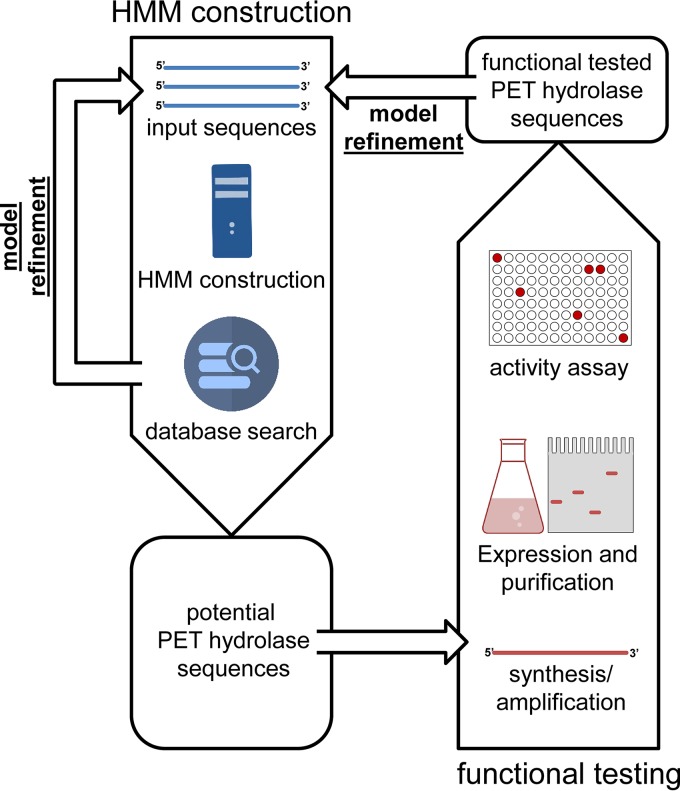
Workflow used in this study to identify and partially characterize novel PET hydrolases from databases and global metagenomes.

In this work, our intention was to mine metagenomes for the detection of novel genes involved in PET degradation and to establish an overview on their taxonomic distribution within the different bacterial phyla. Therefore, we have developed a hidden Markov model (HMM) to search existing genome and metagenome databases for the presence of potential PET hydrolases (Fig. 1; see also Fig. 3B). Using this approach, we identified >500 potential PET hydrolases in the UniProtKB database. In addition, 349 sequence homologs were obtained from several public metagenome data sets deposited on the IMG server, and four of the identified candidate genes were functionally verified. Together, these results imply that PET hydrolase genes are globally distributed in marine and terrestrial metagenomes. Furthermore, we provide evidence that in marine environments, the PET hydrolases originate mainly from the phylum Bacteroidetes and in the terrestrial metagenomes from Actinobacteria.

## RESULTS

### Construction of a hidden Markov model for PET hydrolases.

**FIG 2:**
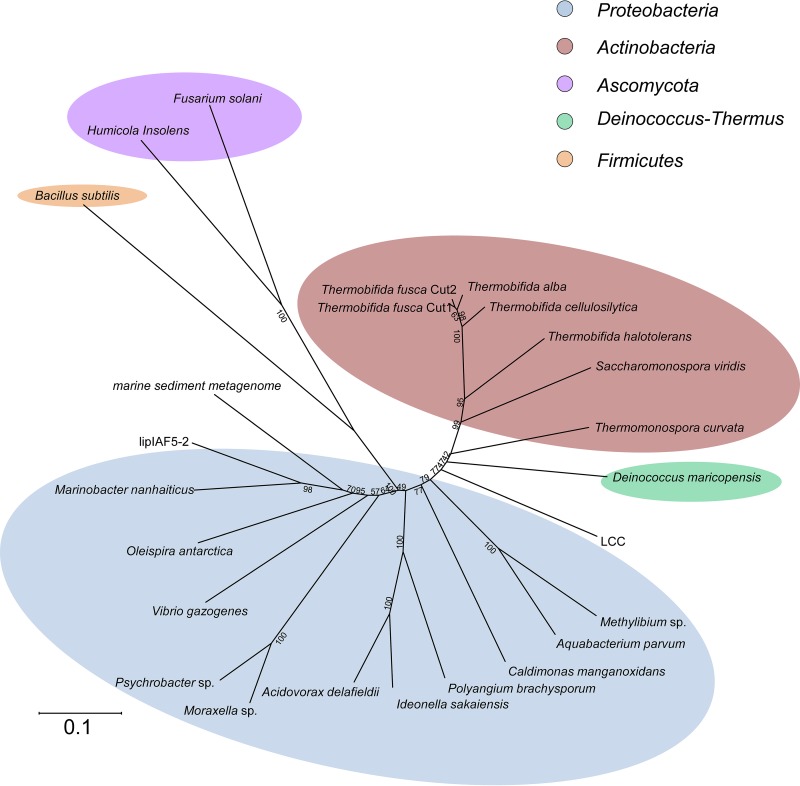
Neighbor-joining tree of manually chosen potential PET hydrolase sequences found in this work. Sequences were obtained from a HMM search in the UniProtKB database and named PET1 to PET13. The tree was calculated using MEGA6. Besides the 13 newly found PET hydrolase sequences (Table S1), 9 already-known PET hydrolases (Table 1) were added to the tree in order to visualize the phylogenetic distribution and similarity of the PET hydrolase sequence homologs.

**FIG 3:**
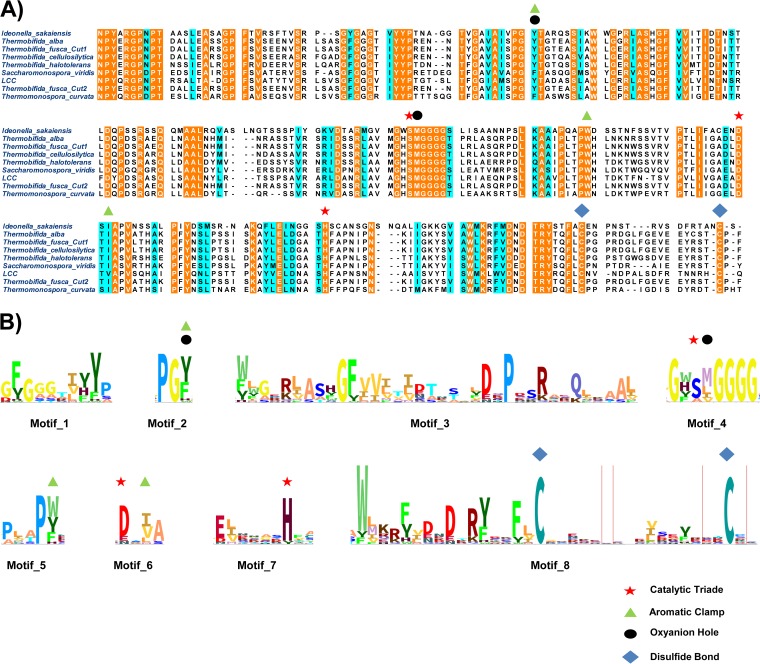
Amino acid sequence alignment of described PET hydrolases. (A) An alignment of the PET hydrolase sequences listed in Table 1 revealed the positions of binding relevant residues and conserved regions. (B) Hidden Markov model (HMM) of PET hydrolase amino acid motifs. The amino acid alignment from panel A was used to calculate a HMM profile. The HMM was consequently visualized as a logo with information content above the background (Skylign; http://skylign.org). Eight sequence motifs are shown in total. Motifs 2 to 8 include amino acids crucial for thermostability, substrate binding, and/or catalytic activity.

Only a few well-characterized PET hydrolases are currently known. The most prominent examples are PET hydrolases from T. fusca and I. sakaiensis (see references in Table 1 and Fig. 2). In this study, we set out to increase knowledge of the diversity of this intriguing group of hydrolases. To identify potential novel PET hydrolases, an amino acid sequence alignment of nine already-known examples was constructed using the T-Coffee multiple sequence alignment server. The enzyme sequences used for the model all have verified activity on PET-based substrates (Table 1). Of the proteins used for the model, seven sequences originated from the phylum Actinobacteria (i.e., sequences 1 to 7; Table 1) and one from the phylum Proteobacteria (Betaproteobacteria) (sequence 8; Table 1), and one sequence (AEV21261) encoded a protein with a metagenomic origin not yet assigned to any phylum (sequence 9, Table 1). A comparison with the well-described PET-active cutinase TfCut2 from Thermobifida fusca allowed the identification of the location of the catalytic triad and other residues that are commonly involved in binding of the substrates (Fig. 3A). Next to the serine of the catalytic triad in every sequence, a methionine residue was found, which is of importance for forming an oxyanion hole together with an aromatic residue. This aromatic residue is also part of an aromatic clamp, together with similar amino acids like tryptophan, tyrosine, histidine, and phenylalanine (17, 21). Terminal cysteine residues are present in all examples and may be important for the thermostability of these enzymes (14, 22). The alignment was used for the construction of a hidden Markov model. For visualization, an HMM logo was created via the Skylign online tool (http://skylign.org/; Fig. 3B). A subsequent visual analysis and conservation prediction using the JS divergence scoring method revealed at least eight conserved regions (Fig. 3B).

An initial HMMER online tool database search with the model against the UniProtKB database revealed a total of 10,854 significant query matches, with a highest bit score value of 441.6. Of these, a subset was chosen that showed a bit score value of >180. In addition, a BLAST search was performed using the newly discovered potential PET hydrolases from the HMM search as initial query sequences against the nonredundant and the metagenomic datasets available at the NCBI database in May 2017. This resulted in the detection of 504 potential PET hydrolase candidate genes. From the obtained homologous sequences, 13 potential PET hydrolase homologs (Fig. 2) were manually chosen due to their sequence similarity to known PET hydrolases (PET1 to PET13). These were used for initial verification and further *in silico* and/or biochemical characterization. These novel predicted PET hydrolases are summarized in Table S1 in the supplemental material, together with their UniProt entries and pfam domain similarities. It was of interest to select mainly nonactinobacterial proteins, in order to diversify the HMM. These 13 sequences were added to the alignment and used for a modified and refined HMM. The 13 initially identified putative PET hydrolases harbor the above-mentioned residues and motifs (listed in Table 2).

**TABLE 2:** Determined search criteria for the identification of PET hydrolase candidate genes in databases

Sequence no.	Search criterion (criteria)^*a*^	Function
1	GxS**M**GGGG	Serine of catalytic triade and methionine for oxyanion hole formation
2	F,Y62	Amino acids for oxyanion hole formation and aromatic clamp
3	W,Y157	
4	I,V180	
5	F,W211	Optional aromatic amino acid for aromatic clamp formation
6	C255 C262	C-terminal cysteine residues for thermostability supporting disulfide bond formation
7	DxDxR(Y)xxF(L)**C**	Conserved sequence prior to first thermostability giving cysteine

a The letter x indicates a nonconserved position within the sequence pattern. Brackets indicate a less conserved position within the sequence pattern. Numbering of amino acids is according to the HMM (see Fig. 3A).

### Classification of PET hydrolases and taxonomic assignments.

An NCBI conserved domain search in the Conserved Domains Database (CDD) database showed that the nine previously known active PET hydrolases (Table 1), as well as the 13 novel (see Table S1) possible homologs, harbor domains belonging to the superfamily of α/β-hydrolases_5 (pfam12695). Of these, only PET9 and PET12 showed specific hits for the superfamily of acetyl xylan esterases (AXE1, pfam05448). In addition to the specific hits, several nonspecific domain hits were obtained (Table S1).

**FIG 4:**
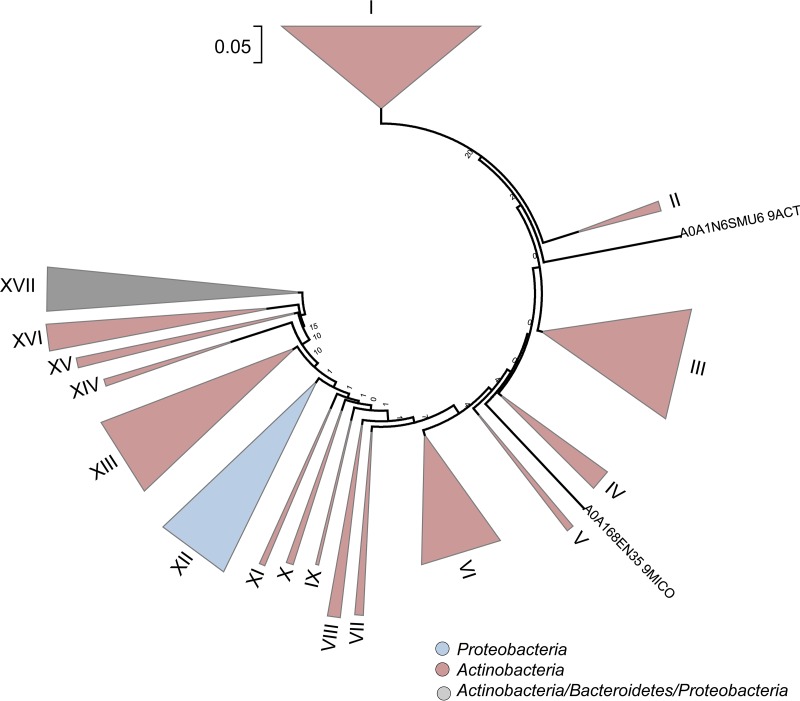
Classification and phylogenetic tree of 504 novel and potential PET hydrolases obtained by HMM searches. Sequences were obtained from the UniProtKB database. A total of 504 sequences identified with the constructed HMM and having a bit score of >180 were visualized, of which the sequences of PET1 to PET13 (Table S1), as well as 9 already described PET hydrolases (Table 1), represent a subset of the newly found potential enzymes.

A further alignment and subsequent tree calculation with the above identified 504 potential PET hydrolases allowed the assignment of all enzymes in 17 subclasses (Fig 4). Interestingly, two sequences (UniProt numbers A0A1N6SMU6 and A0A168EN35) did form individual subclusters and could not be assigned to other clusters. The majority of the subclasses were mainly affiliated with Actinobacteria, and only one subclass (XII) was associated with Proteobacteria. For the subclass XVII, no clear assignment was possible. The Thermobifida PET hydrolase sequences are clustered within subcluster XV, together with the PET hydrolase from Saccharomonospora. The leaf compost cutinase (LCC) sequence was found in subcluster XI and the Thermomonospora curvata sequence is located in group XII. The PET hydrolase from Ideonella sakaiensis is located in subcluster VI.

### Experimental verification of the HMM and characterization of selected novel PET hydrolases.

**FIG 5:**
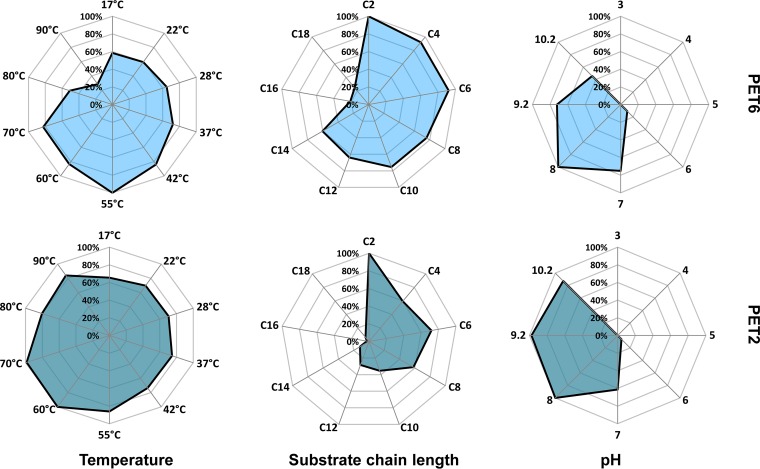
Biochemical characterization of PET2 and PET6 with different *p*NP substrates. Data obtained with a *p*NP assay are shown in net diagrams for PET2 and PET6. Substrate preferences, temperature optimum, and pH optimum were tested. All tests besides substrate preferences were carried out with *p*NP octanoate.

Since the bioinformatic approach only delivered potential PET hydrolases enzymes, we initiated work to verify a small number of the identified candidate genes with respect to their function. Therefore, we chose the enzymes PET 2, 5, 6, and 12 (Table S1). The respective genes were either synthesized or amplified from genomic DNA using vectors and primers (as outlined in Tables 3 and 4) and were cloned into the expression vectors. Initial tests indicated that all genes coded for active enzymes. On agar plates containing PET nanoparticles or polycaprolactone (PCL) (9, 24), all active clones produced halos after overnight incubation. They were compared to the PET hydrolase from Thermobifida fusca as a positive control (see Fig. S1 in the supplemental material). PCL was used as a model substrate, as hydrolysis of this compound indicates possible activities on the more complex PET. From these active enzymes, we chose two enzymes for more detailed biochemical characterization. These were the two enzymes PET 2 and PET 6. PET 2 was derived from a marine metagenomics data set (25), and PET 6 was derived from Vibrio gazogenes strain DSM-21264 (26). After successful expression and purification of the two enzymes in sufficient amounts, the obtained enzymes were further characterized using *para*-nitrophenyl esters (*p*NP esters) (Fig. 5; see also Fig. S2 and S3 in the supplemental material). Both enzymes showed best activity against *p*NP esters (C_2_ to C_4_), but were able to convert long-chain (>C_10_) substrates as well. Their temperature optimums were 55°C and 70°C for PET6 and PET2, respectively. Remarkably, PET2 retained 80% of its relative activity at 90°C after incubation for >5 h. Both enzymes preferred alkaline pH values of 8 to 9. Rubidium at a concentration of 1 mmol/liter had a strong effect in the case of PET2 by increasing the activity by 50%. A similar but significantly smaller effect was observed in the case of PET6. Both enzymes showed reduced hydrolytic activity in the presence of 5% SDS, 10 mM phenylmethylsulfonyl fluoride (PMSF), and 30% acetonitrile. Additional high-performance liquid chromatography (HPLC) analyses confirmed the above findings for PET2. In tests using 14 mg of amorphous PET foil as the substrate, 100 μg of PET 2 was able to release 900 μM terephthalic acid after 24 h of incubation (see Fig. S4 in the supplemental material).

**TABLE 3:** Bacterial strains and plasmids used in this work

Strain or plasmid	Property(ies)^*a*^	Reference or source
Strains		
E. coli DH5α	*supE44* Δ*lacU*169 (Ф80 *lacZ* Δ*M15*) *hsdR17 recA1 endA1 gyrA*96 *thi-1 relA1*	23
E. coli BL21(DE3)	F^−^ *ompT hsdS* B (r_B_^−^ m_B_^−^) *gal dcm* λDE3	Novagen/Merck (Darmstadt, Germany)
E. coli T7SHuffle Express	*fhuA2 lacZ*::*T7 gene1 [lon] ompT ahpC gal λatt*::*pNEB3-r1-cDsbC (SpecR*, *lacIq) ΔtrxB sulA11 R(mcr-73*::miniTn*10*-TetS*)2 [dcm] R(zgb-210*::Tn*10*-TetS*) endA1 Δgor Δ(mcrC-mrr)114*::IS*10*	NEB (Frankfurt am Main, Germany)
Deinococcus maricopensis DSM-21211	Type strain	DSMZ (Braunschweig, Germany)
Vibrio gazogenes DSM-21264	Type strain	DSMZ (Braunschweig, Germany)
Polyangium brachysporum DSM-7029	Type strain	DSMZ (Braunschweig, Germany)
Plasmids		
pET21a(+)	Expression vector, *lacI*, Amp^r^, T7 *lac* promoter, C-terminal His_6_-tag coding sequence	Novagen/Merck (Darmstadt, Germany
pET28a(+)	Expression vector, *lacI*, Amp^r^, T7 *lac* promoter, C-terminal His_6_-tag and N-terminal coding sequence	Novagen/Merck (Darmstadt, Germany
pEX-A2	Cloning vector, Amp^r^, P_lac_*lacZ*, pUC *ori*	Eurofins MWG Operon (Ebersberg, Germany)

a Amp^r^, ampicillin resistance.

**TABLE 4:** Primers used in this work

Primer	Sequence (5′ → 3′)	Length (bp)	*T_m_* (°C)^*a*^	Source
T7 promoter	TAATACGACTCACTATAGGG	20	53.2	Eurofins MWG (Ebersberg, Germany)
T7 terminator	CTAGTTATTGCTCAGCGGT	19	54.5	Eurofins MWG (Ebersberg, Germany)
PET5_for	CGCCGCCATATGAATAAATCTATTCTAAAAAAACTCTC	38	68	This work
PET5_rev	CGATTCGGCGGCCGCGTAATTACATGTGTCACGG	34	77	This work
PET6_for	CGTAGTCATATGGTACCGTGTTCGGACTG	29	69	This work
PET6_rev	CAGCGGCCGCCTAATAGTAACTACAGTTGTCTCG	34	73	This work
PET12_for	CGCCATATGCAGACCAACCCCTACCAGCGAGGCCC	35	80	This work
PET12_rev	CTTGCGGCCGCTCAGTACGGGCAGCTCTCGCGGTACTCC	39	84	This work

a *T*_*m*_, melting temperature.

Altogether, the data presented above indicate that the developed search algorithm is useful for the identification of novel and functionally active PET hydrolases from single genomes and metagenomes.

### Global distribution of PET hydrolases and their significance in marine and terrestrial environments.

**FIG 6:**
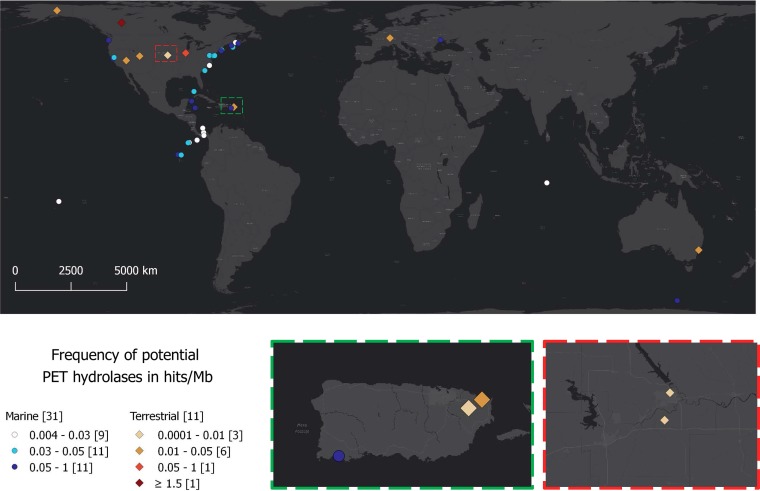
Global distribution of PET hydrolases in available metagenomes. Potential PET hydrolase containing metagenomes were visualized on a world map, using circles for marine and triangles for terrestrial metagenomes. Blue and red color shading indicates the frequency of PET hydrolase genes in hits/Mb for marine and terrestrial metagenomes, respectively. Red and green boxes magnify regions with overlapping spots (sample sites). (Map constructed using qGIS Desktop 2.18.5 [http://www.qgis.org].)

After the successful construction of a reliable HMM and the identification, as well as partial characterization, of new PET hydrolases, we asked if these enzymes could be identified on a global level, and if so, to what extent. To evaluate the environmental distribution of sequences encoding PET hydrolases, the data from 108 marine and 25 terrestrial metagenomes were taken into account and downloaded from the IMG database (27) (see Table S2 in the supplemental material). Criteria for the selection of marine data included sample depth (maximum 2 m), assembly status, global distribution of sample locations, and size and availability of the data set. The same criteria, except for the sample depth, were chosen for terrestrial metagenomes. The size of the assembled metagenome data in the case of marine metagenomes ranged from 10.85 Mb up to 7.99 Gb. In the case of terrestrial metagenomes, the number of assembled bases ranged from 58 Mb to 9.2 Gb. The modified HMM was used to find PET hydrolase homologs in the sequence data of those metagenomes on a global scale. The searches identified possible PET hydrolase homologs in 31 marine and 11 terrestrial metagenomes. A total of 349 hits was observed for these 42 samples. The number of hits per sample was normalized, calculated as hits per Mb, and visualized on a global map representing the geographical location as well as the frequency of PET hydrolase homologs (Fig. 6). Within the marine and terrestrial metagenomes, PET hydrolase frequencies ranged from 0.004 to 0.92 hits/Mb and 0.0001 to 1.513 hits/Mb, respectively.

The combined genome sizes of terrestrial metagenomes are nearly 2.5-fold higher than those of the marine metagenomes, and they harbor 157 PET hydrolase homologs in average. In contrast, the marine metagenomes harbor an average of 42 PET hydrolases. The terrestrial metagenome with the highest abundance of potential PET hydrolases contains 135 sequence hits and was derived from the sediment core of a heavy oil reservoir in Canada (IMG genome number 3300001197). In the case of the marine metagenomes, the maximum was 31 hits, found within the metagenome data of a sample from the Delaware coast in the United States (Fig. 6).

**FIG 7:**
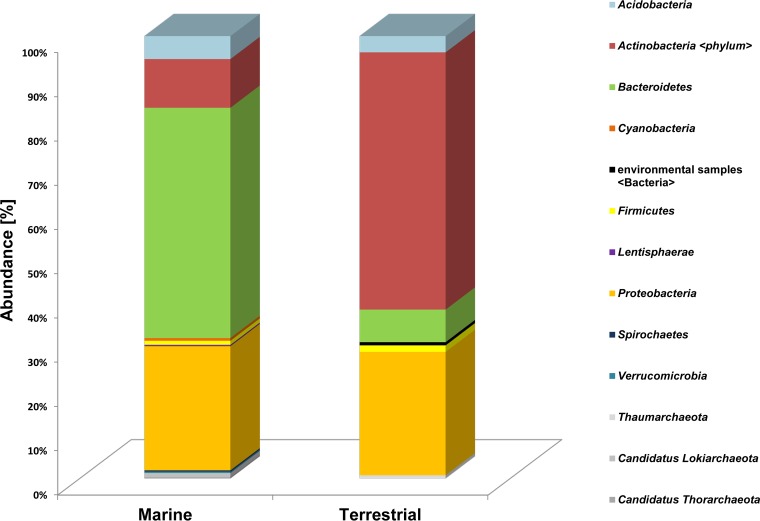
Phylogenetic affiliation of 349 predicted PET hydrolases from 31 marine and 11 terrestrial metagenomes. Colored and stacked bars represent the number of hits per phylum. Data were normalized per Mb of assembled DNA for the analyzed samples.

We further observed that within the terrestrial habitats, the Actinobacteria were the main hosts for the terrestrial-derived enzymes. However, in the marine samples, most predicted PET hydrolases originated from the phylum of Bacteroidetes (Fig. 7). Bacteroidetes sequences in the marine samples were affiliated with 43% of all hits. The phylum Proteobacteria was the second-most abundant in both data sets, with 23% of hits in marine and 20% in terrestrial data (Fig. 7).

## DISCUSSION

In this work, we developed a search algorithm that allows the *in silico* identification of PET hydrolase gene candidates from genomes and metagenomes. Altogether, we were able to identify 504 novel possible enzyme candidates in the UniProtKB and nonredundant RefSeq databases and the metagenomic database available in the NCBI database. In addition, we identified 349 candidate genes and enzymes from marine and terrestrial metagenomes available at the IMG platform. This is by far the largest collection of PET hydrolase enzyme candidates currently available. A first classification of the PET hydrolases derived from UniProtKB/GenBank due to their protein sequence similarities and occurrence of conserved homologs enabled the formation of 17 enzyme clusters in this work (Fig. 4). An additional search of global metagenomes revealed that the PET hydrolases occur in both marine and terrestrial habitats. However, the frequencies (hits per Mb) are comparably low. The lowest hit rate was observed for a metagenome from a Kansas (United States) prairie soil sample and the highest hit rate was observed for a metagenome derived from a heavy oil reservoir sediment core. Since we included over 100 metagenomes in this analysis, the data give a reliable picture of the overall occurrence of these enzymes, but do not allow estimations on the expression of these genes in the native environment. However, the overall low gene frequencies might suggest that bacterial evolution has not yet allowed the spreading of this trait. This also implies that the overall degradation potential of the oceans is rather low compared to that of other habitats and enzymes involved in the breakdown of natural polymers like starch or cellulose (28, 29).

We verified the usefulness of the developed algorithm by cloning four novel PET hydrolase genes and expressing them heterologously in E. coli. All enzyme clones were active and supported the notion that the developed search algorithm is useful. The enzyme properties of the newly characterized PET hydrolases fit well into the overall picture of known PET hydrolyzing enzymes, with an optimum pH at slightly alkaline values and a preference for substrates with a short chain length (14) (30). Overall, these novel enzymes revealed comparable activities to those already previously characterized (see Table 1 and references therein).

To our surprise, both enzymes characterized in more detail (PET2 and PET6) showed traits of thermostability (Fig. 5) (25). PET2 was stable up to 90°C, with measurable residual activity of more than 50%. This is more stable than the LCC derived from a compost metagenome (14).

All of the newly identified PET hydrolases originated mainly from three bacterial phyla, Proteobacteria, Actinobacteria, and Bacteroidetes. Within this framework, it is notable that the Bacteroidetes have so far not been associated with PET degradation, but Bacteroidetes species have been described as very potent degraders of other polymers, and they harbor a multitude of hydrolases and binding modules (31–33).

The restriction of PET hydrolases to a few bacterial phyla could indicate that this metabolic capability has only rather recently been evolved and is thus limited to a very few phylogenetic groups. The observation here that in the marine habitat the phylum Bacteroidetes is the main host of PET hydrolases is new and intriguing for several reasons. First, using classical searches and biochemical characterization, the Actinobacteria and Proteobacteria were considered to be the main hosts for these enzymes (Table 1). Second, the searches in UNIProtKB and other databases implemented on the NCBI website underlined the presence of PET hydrolases in the phyla Actinobacteria and Proteobacteria. Only when we extended our search for metagenomes of mainly noncultivated bacterial phyla did we identify the Bacteroidetes as the main hosts for these enzymes in the marine environment.

The recent findings on PET hydrolases described in this publication will significantly extend the knowledge of these enzymes and provide promising candidates for biotechnological applications. In summary, the over 800 enzyme candidates identified in this work will build the basis for a global repository and database of this urgently needed enzyme class.

## MATERIALS AND METHODS

### Bacterial strains, plasmids, and primers.

Bacterial strains, plasmids, and primers used in this study are listed in Tables 3 and 4. If not otherwise mentioned, Escherichia coli clones were grown in LB medium (1% tryptone/peptone, 0.5% yeast extract, and 1% NaCl) supplemented with appropriate antibiotics (25 μg/ml kanamycin or 100 μg/ml ampicillin) at 37°C for 18 h.

### Databases used in this study and bioinformatic analysis.

Nucleotide and amino acid sequences of putative PET hydrolases were acquired from databases integrated into the NCBI (https://www.ncbi.nlm.nih.gov/), UniProt (http://www.uniprot.org/) and the Joint Genome Institute (JGI) IMG (https://img.jgi.doe.gov/) websites (34), (27, 35). Sequences were compared to others deposited in the NCBI databases using BLAST alignment tools (36). Amino acid sequence HMM search was carried out using the HMMER (http://hmmer.org/) webpage or a local version of the software (v3.1b2) with downloaded data sets. Structural information on the enzymes was retrieved from the RCSB-PDB (37) database.

Sequence data were processed using BioEdit and the Clone Manager suite version 9 (Sci-Ed Software, Denver, USA). Neighbor-joining phylogenetic trees based on amino acid sequence alignments were constructed using MEGA6 (38). Nine known and activity-confirmed bacterial PET hydrolase sequences were obtained from NCBI, aligned with T-Coffee (39) and manually revised. Afterwards, the alignment was used to construct a profile HMM with the “hmmbuild” function of the HMMER package (http://hmmer.org/). After the identification of PET hydrolase homologs, the obtained sequences were included in the above-mentioned alignment and the HMM was refined. An HMM logo was visualized using the Skylign online tool (40). Metagenomic data were downloaded from the IMG database using a Globus endpoint and were further analyzed using “hmmsearch” from the HMMER package. Phylogenetic assignment was done via a local diamond-blast search (41) against the nonredundant protein database (36) and subsequent analysis with MEGAN6 (42). The map representing the frequency and geographical distribution of PET hydrolases in metagenomes (Fig. 6) was constructed using qGIS Desktop 2.18.5 (http://www.qgis.org/).

### Cloning and heterologous expression of PET2, PET5, PET6, and PET12 in Escherichia coli T7-SHuffle.

 Cloning of PET hydrolase genes into the expression vectors pET21a(+) and pET28a(+) was accomplished after amplification of genomic DNA using specific primer pairs with underlined homolog regions to the vector or restriction sites. The sequence of PET2 was obtained from NCBI (GenBank accession number ACC95208) and synthesized after codon usage optimization for E. coli (MWG Eurofins, Germany). Obtained DNAs were cloned into expression vectors, and the constructs were transformed into E. coli T7-SHuffle cells. The cultures were grown aerobically in autoinduction medium (ZYM-5052) (43) containing 100 μg/ml ampicillin and 25 μg/ml kanamycin for pET21a(+) and pET28a(+), respectively, at 37°C until they reached an optical density at 600 nm (OD_600_) of 1.0. The proteins harboring a C- or N-terminal histidine tag were expressed afterwards at 17°C for 16 to 20 h. The cells were harvested and lysed with pressure using a French press. Afterwards, the proteins were purified with nickel-ion affinity chromatography using nickel-nitrilotriacetic acid (Ni-NTA) agarose (Qiagen, Hilden, Germany) and analyzed by SDS-PAGE. The elution buffer was exchanged against 0.1 mM potassium phosphate buffer pH 8.0 in a 10 kDa Amicon tube (GE Health Care, Solingen, Germany).

### Biochemical characterization of PET2 and PET6.

For activity tests, both enzymes were assayed using purified recombinant protein. Unless otherwise indicated, the enzymes were added to a substrate solution containing 190 μl of either 0.2 M sodium phosphate buffer or 0.1 M Tris-HCl, with a defined pH between 7 and 8 and 0.5 mM *p*NP substrate dissolved in isopropanol. Incubation time ranged from 15 to 30 min. As substrates, we tested *p*NP esters with chain lengths of C_2_, C_4_, C_6_, C_8_, C_10_, C_12_, C_14_, C_16_, and C_18_. After incubation at a defined temperature, the color change from colorless to yellow was measured at 405 nm in a plate reader (Biotek, Winooski, USA). All samples were measured in triplicate. For determination of the optimal temperature, samples were incubated between 17°C and 90°C for 15 min. The impact of pH conditions on the activity of each enzyme was measured in citrate phosphate (pH 3.0, 4.0, and 5.0), potassium phosphate (pH 6.0, 7.0, and 8.0), and carbonate bicarbonate buffer (pH 9.2 and 10.2). The influence of possible cofactors, solvents, detergents, and inhibitors was assayed at different concentration levels. After 1 h of incubation in the presence of the substances described below, the residual activity was determined after 15 min incubation at optimal temperature with *p*NP-octanoate and optimal pH. The possible cofactors Ca^2+^, Co^2+^, Cu^2+^, Fe^3+^, Mg^2+^, Mn^2+^, Rb^2+^, and Zn^2+^, with final concentrations of 1 and 10 mM, were used. To determine the solvent stability, dimethyl sulfoxide (DMSO), isopropanol, methanol, dimethylformamide (DMF), acetone, acetonitrile, and ethanol, with final concentrations of 10% and 30% (vol/vol) were added to the reaction. Detergent stability was assayed with SDS, Triton X-100, and Tween 80 at 1% and 5% (wt/vol, vol/vol) concentration. The inhibitory effects of EDTA, dithiothreitol (DTT), and PMSF were tested at 1 and 10 mM concentration. Substrate analyses using the HPLC LaChrom Elite system from Hitachi (Tokyo, Japan) with a Lichrospher 100 RP-18e column (VWR International GmbH, Darmstadt, Germany), consisting of 5-μm diameter particles, were done as previously published (14). A 14-mg low-crystallinity PET film (Goodfellow GmbH, Bad Nauheim, Germany) was used as the substrate. For enzymatic hydrolysis, up to 50 μg of protein was incubated at 60°C with continuous shaking at 500 rpm. As a mobile phase, acetonitrile (A) and water with 0.1% trifluoroacetic acid (TFA) (B) were used in a isocratic method with 20% acetonitrile (A). The reaction buffer was 0.1 M Tris-HCl (pH 7.5), with an injection volume of 99 μl. Detection was performed at 241 nm.

## Supplementary Material

Supplemental material
