# Supplementary material for: New Insights into the Function and Global Distribution of Polyethylene Terephthalate (PET)-Degrading Bacteria and Enzymes in Marine and Terrestrial Metagenomes
Source: Appl Environ Microbiol. 2018 Apr 2;84(8):e02773-17. doi: 10.1128/AEM.02773-17 (PMC5881046; doi:10.1128/AEM.02773-17)
Supplement: Supplemental material [file supp_84_8_e02773-17__index.html]

New Insights into the Function and Global Distribution of Polyethylene Terephthalate (PET)-Degrading Bacteria and Enzymes in Marine and Terrestrial Metagenomes — Supplemental material 

# New Insights into the Function and Global Distribution of Polyethylene Terephthalate (PET)-Degrading Bacteria and Enzymes in Marine and Terrestrial Metagenomes

## Supplemental material

- Supplemental file 1 -

  PET and PCL plate assay (Fig. S1); biochemical characterization of PET2 (Fig. S2) or PET6 (Fig. S3) with different *p*NP substrates; chromatographic analysis of PET degradation by PET2 (Fig. S4); newly identified PET hydrolases (Table S1); marine and terrestrial metagenomes used for PET hydrolase search (Table S2).

  PDF, 484K
