# Supplementary material for: New Insights into the Function and Global Distribution of Polyethylene Terephthalate (PET)-Degrading Bacteria and Enzymes in Marine and Terrestrial Metagenomes
Source: Appl Environ Microbiol. 2018 Apr 2;84(8):e02773-17. doi: 10.1128/AEM.02773-17 (PMC5881046; doi:10.1128/AEM.02773-17)
Supplement: Supplemental material [file AEM.02773-17_zam008188431s1.pdf]

SUPPLEMENTAL FIGURES

FIGURE S1. PET and PCL plate assay to determine activity of PET2, 5 6 and 12. Activity against PET and PCL was tested with purified enzyme. Plates were incubated at room temperature over night or at 50°C over night for PCL and PET respectively. Formation of a halo confirmed activity.

FIGURE S2. Biochemical characterization of PET2 with different *p*NP-substrates. Data obtained with a *p*NP-assay are shown in net diagrams for PET2. Co-factor dependency, inhibitory substance effect, detergent effect and the effect of different solvents were tested. All tests were carried out with *p*NP-octanoate.

FIGURE S3. Biochemical characterization of PET6 with different *p*NP-substrates. Data obtained with a *p*NP-assay are shown in net diagrams for PET6. Co-factor dependency, inhibitory substance effect, detergent effect and the effect of different solvents were tested. All tests were carried out with *p*NP-octanoate.

FIGURE S4. Chromatographic analysis of PET degradation by PET2. The degradation of PET foil was observed after 24h of incubation at 60°C. In order to assign the degradation product to the obtained peaks TPA, BHET and MHET served as reference substances.

SUPPLEMENTAL TABLES

Table S1: Newly identified PET hydrolases (PET1-PET13). The newly identified PET hydrolases in this work were named PET1-PET13. Besides unspecific hits, the CDD search revealed several specific conserved domain similarities to pfam superfamilys.

Table S2: Marine and terrestrial metagenomes used for PET hydrolase search. Data were obtained from IMG. Besides IMG genome ID and sample name, the HMM obtained hits and PET hydrolase frequencies are shown in hits/Mb.

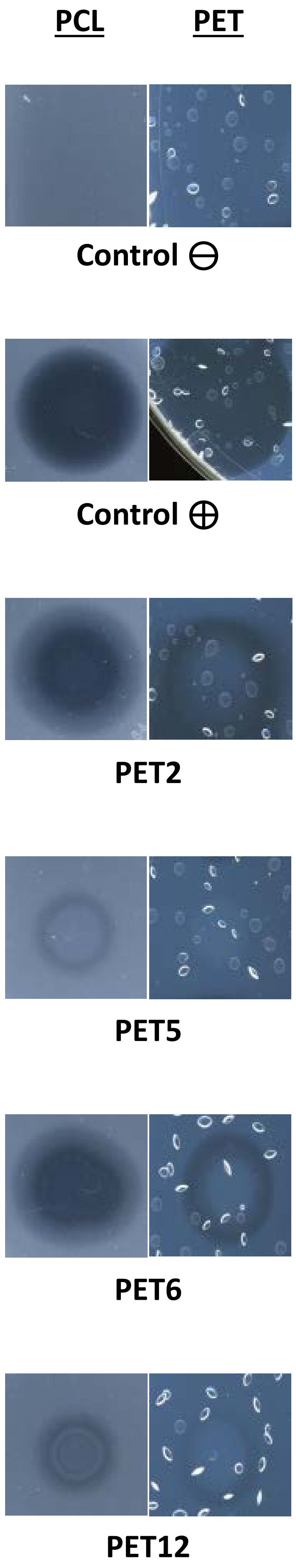

FIGURE S1. PET and PCL plate assay to determine activity of PET2, 5 6 and 12. Activity against PET and PCL was tested with purified enzyme. Plates were incubated at room temperature over night or at 50°C over night for PCL and PET respectively. Formation of a halo confirmed activity.

PET2

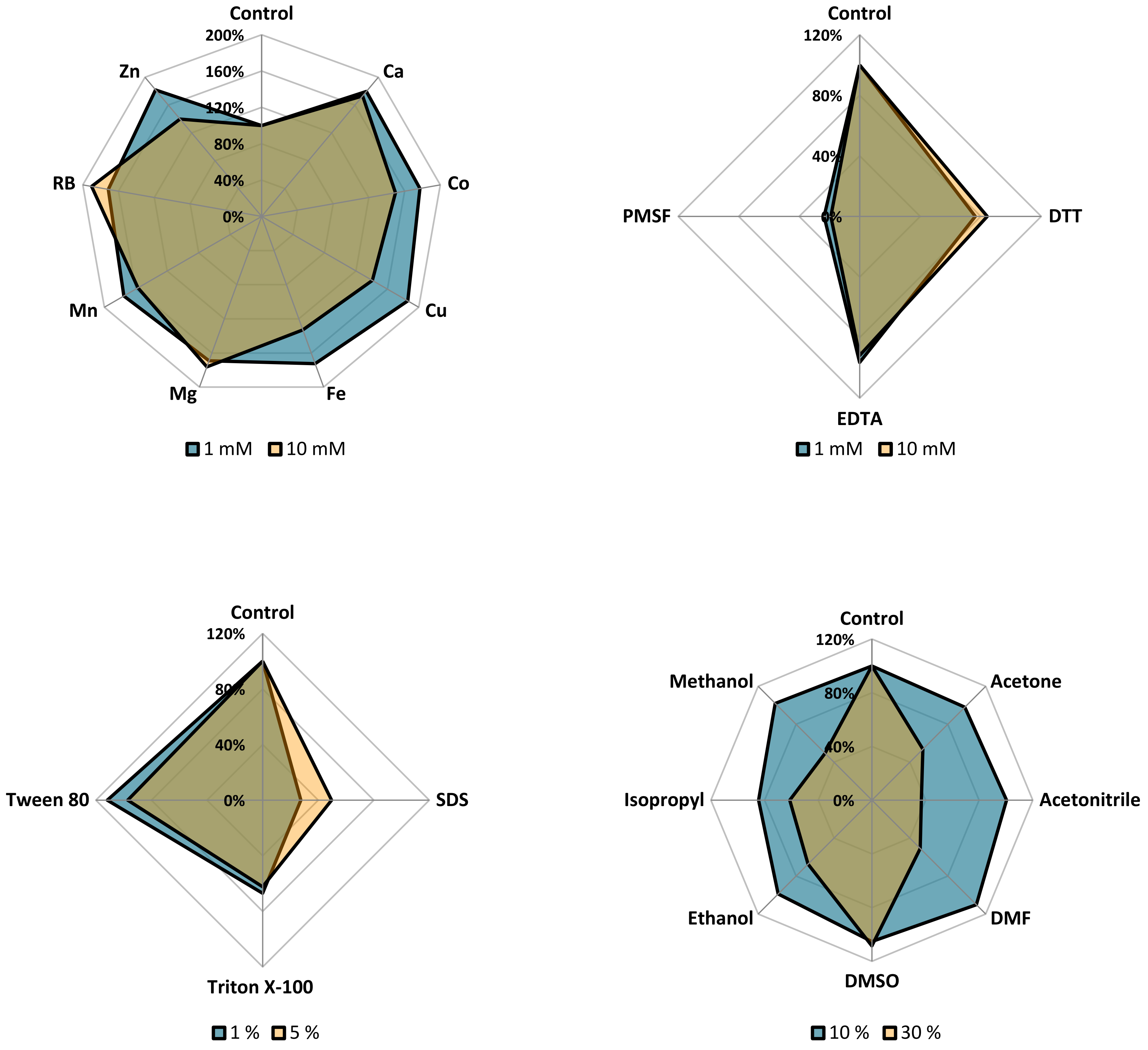

FIGURE S2. Biochemical characterization of PET2 and PET6 with different *p*NP-substrates. Data obtained with a *p*NP-assay are shown in net diagrams for PET2. Co-factor dependency, inhibitory substance effect, detergent effect and the effect of different solvents were tested. All tests were carried out with *p*NP-octanoate

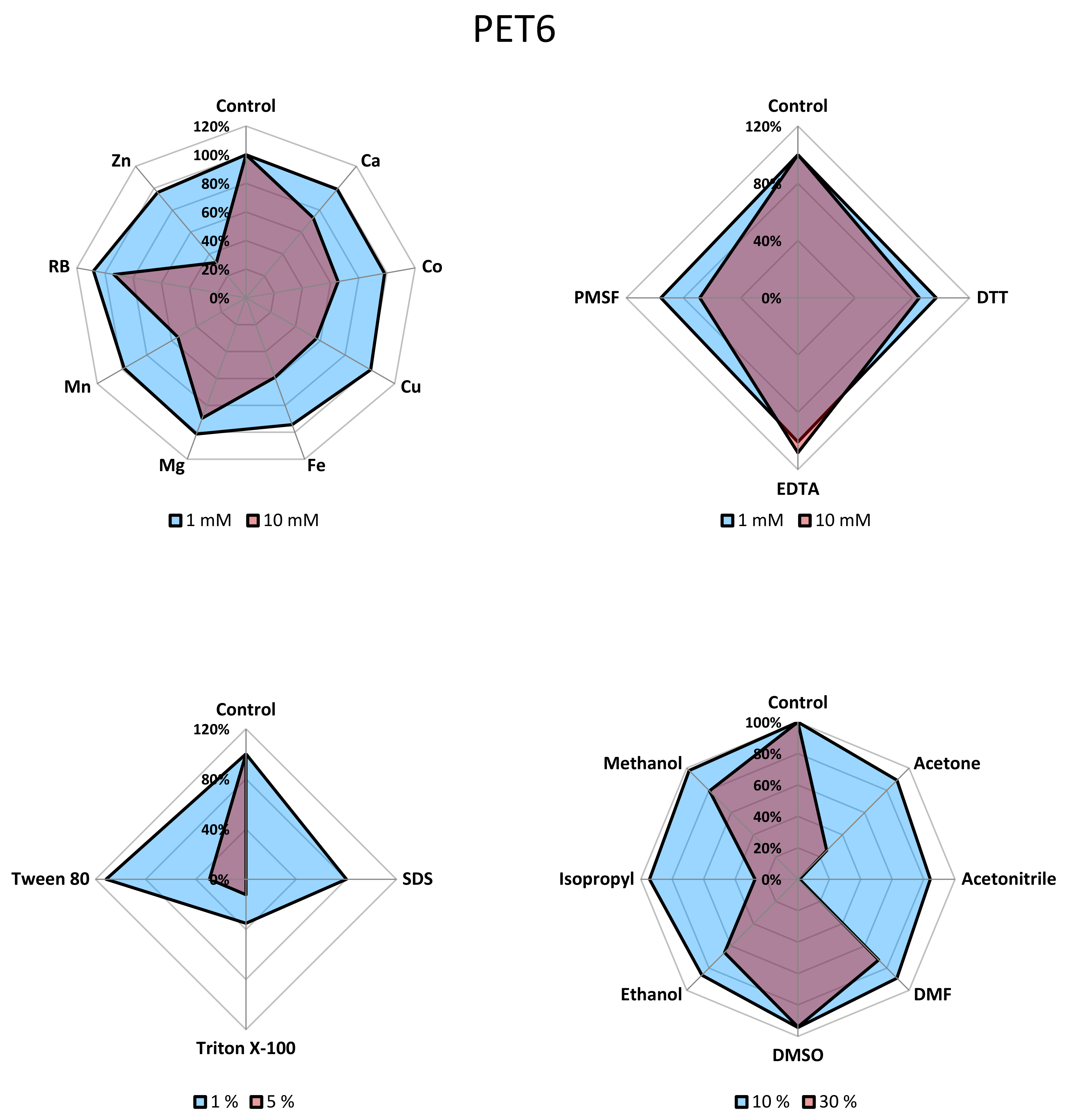

FIGURE S3. Biochemical characterization of PET6 with different *p*NP-substrates. Data obtained with a *p*NP-assay are shown in net diagrams for PET6. Co-factor dependency, inhibitory substance effect, detergent effect and the effect of different solvents were tested. All tests were carried out with *p*NP-octanoate.

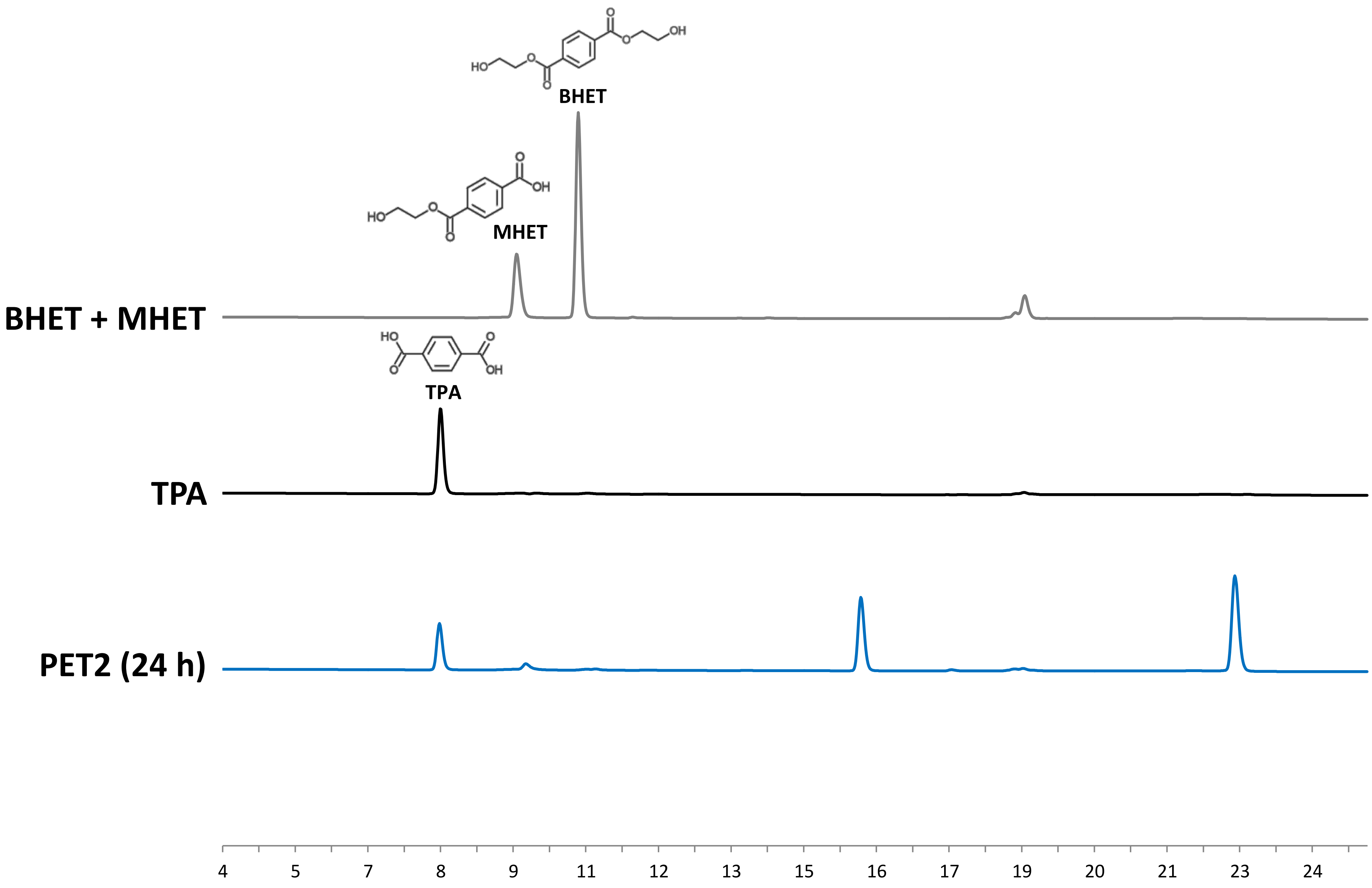

FIGURE S4. Chromatographic analysis of PET degradation by PET2. The degradation of PET foil was observed after 24h of incubation at 60°C. In order to assign the degradation product to the obtained peaks TPA, BHET and MHET served as reference substances.

PET#	UniProt_Entry	Gene name	Organism	Phylum	Pfam / CDD ID
1	E8U721	Deima_1209	*Deinococcus maricopensis* (strain DSM 21211)	Deinococcus-Thermus	cl26327, pfam01738, pfam12695, pfam00561, pfam05448, cl27026, pfam12146, cl21494, cl26327, pfam01738, pfam12695, pfam00561, pfam05448, cl27026, pfam12146
2	C3RYL0	lipIAF5-2	uncultured bacterium	n.d	cl27026, pfam00326, pfam01738, pfam05448, cl21494, cl27026, pfam00326, pfam01738, pfam05448
3	A0A0F9X315	LCGC14_0200860	marine sediment metagenome	n.d	cl27026, pfam01738, pfam05448, cl21494, cl27026, pfam01738, pfam05448
4	N6VY44	J057_15340	*Marinobacter nanhaiticus* D15-8W	Proteobacteria	pfam01738, pfam00326, cl21494, , pfam01738, pfam00326
5	R4YKL9	lipA OLEAN_C07960	*Oleispira antarctica* RB-8	Proteobacteria	n.d
6	UPI0003945E1F	n.d	*Vibrio gazogenes*	Proteobacteria	cl27026, pfam00326, pfam05448, cl21494, cl27026, pfam00326, pfam05448
7	Q8RR62	pbsA	*Acidovorax delafieldii*	Proteobacteria	n.d
8	P19833	lip1 L1	*Moraxella* sp. (strain TA144)	Proteobacteria	cl27026, pfam00326, pfam01738, pfam05448, cl21494, cl27026, pfam00326, pfam01738, pfam05448
9	A0A0D4L7E6	est	*Psychrobacter* sp. PAMC 21119	Proteobacteria	cl27026, pfam00326, pfam01738, pfam05448, cl21494, cl27026, pfam00326, pfam01738, pfam05448
10	UPI00064655D2	n.d	*Methylibium* sp.	Proteobacteria	pfam12740, cl21494, pfam12740
11	UPI0003660256	n.d	*Caldimonas manganoxidans*	Proteobacteria	cl26327, pfam01738, pfam00756, pfam12146, pfam03403, cl21494, cl26327, pfam01738, pfam00756, pfam12146, pfam03403

12	A0A0G3BI90	AAW51_2473	*[Polyangium] brachysporum*	Proteobacteria	cl27026, pfam00326, pfam01738, pfam05448, pfam12695, cl21494, cl27026, pfam00326, pfam01738, pfam05448, pfam12695
13	A0A1F4G492	A2711_12500	*Burkholderiales bacterium*	Proteobacteria	cl21494, cl21494, pfam12740, pfam12695, pfam00756, pfam01738, pfam07224, pfam03403

Terrestrial metagenomes

IMG Genome ID	Genome Name / Sample Name	Latitude	Longitude	Genome Size * assembled	Gene Count * assembled	#Hits	Hits/GB
2070309004	Green-waste compost microbial communities at University of California, Davis, USA, from solid state bioreactor - Luquillo Rain Forest, Puerto Rico	18.311389	-65.8375	281407472	781631	1	0.004
2119805012	Soil microbial communities from sample at FACE Site NTS_067 Nevada Test Site (NTS_067)	36.766667	-115.95	390792833	1036364	14	0.036
2140918008	Permafrost microbial communities from permafrost in Bonanza Creek, Alaska - Bog_all (Bog_all_CLC)	64.7	-148.3	410522816	696868	5	0.012
2162886008	Soil microbial communities from Puerto Rico rain forest, that decompose switchgrass - Feedstock-adapted consortia SG + Fe (SG + Fe, May 2011 assembly)	18.3724	-65.7166	154120208	193491	2	0.013
2209111000	Soil microbial communities from Colorado Plateau, Greene Butte sample - Dark Crust, Colorado Plateau, Green Butte (Dark Crust, Colorado Plateau, Green Butte June 2011 assem)	38.714972	-109.692944	396718301	1058313	7	0.018
3300000596	Amended soil microbial communities from Kansas Great Prairies, USA - Total DNA no BrdU F1.4TC (Kansas native prairie Total DNA no BrdU F1.4TC, April 2012 Assem)	39.100992	-96.608258	118917357	353727	1	0.008
3300000956	Soil microbial communities from Great Prairies - Kansas, Native Prairie soil (Kansas, Native Prairie soil, Sept. 2012 Assem JGI Velvet)	39.214012	-96.585283	9280824611	31876498	1	0.000
3300001197	Wastewater microbial communities from Syncrude, Ft. McMurray, Alberta - Microbes from Sediment core from a heavy oil	56.04	-118.13	89229498	204944	135	1.513

	reservoir,Alberta Canada Inniskillen 614.3 (Inniskillen 614.3: 454+illumina sequencing assembly)						
3300002468	Deep subsurface microbial communities from Mt. Terri Underground Rock Laboratory, Switzerland - 10_samples_coassembly (concoct_output_2nd_run)	47.379	7.1648	143773111	146707	2	0.014
3300005258	Microbial communities on the surface of bentonite enhanced biochar (D2B)	-33.917926	151.235347	162951842	282734	4	0.025
3300005260	Microbial communities on the surface of kaolinite enhanced biochar from soil with fertiliser in Sydney, Australia (F2B)	-33.917926	151.235347	308772089	487956	5	0.016
2044078003	Miscanthus field bulk soil microbial communities from University of Illinois Energy Farm, Urbana, IL (Bulk soil sample from field growing Miscanthus x giganteus)	40.109	-88.204	58125848	165002	5	0.086
2067725009	Permafrost microbial communities from central Alaska, USA - Permafrost field sample	65.7906	-149.9102	9656814	13277	0	0.000
2189573024	Echo Passage metagenome	31.837801	-110.350292	138499921	365407	0	0.000
3300000044	Arabidopsis rhizosphere microbial communities from the University of North Carolina - sample from Arabidopsis soil old (Arabidopsis soil old, Nov 2011 assem)	35.9	-79.05	92517998	280064	0	0.000
3300000597	Forest soil microbial communities from Amazon forest - 2010 replicate II A1 (Amazon Forest 2010 replicate II A1, April 2012 Assem)	-10.171667	-62.7875	321952212	768309	0	0.000
3300000793	Forest soil microbial communities from Amazon forest - 2010 replicate II A001 (Amazon Forest 2010 replicate II A001, March 2012 Assem)	-10.171667	-62.7875	297027237	799655	0	0.000
3300000825	Subaerial biofilm microbial communities from sulfidic caves, Italy, that are extremely acidic - Acquasanta AS5 (Draft assembly, stringent	42.755	13.411118	8590020	17561	0	0.000

	(mi 99 ml 60 rip))						
3300001490	Fosmid Clones Derived from Amazon Forest Soil Microbial Communities (Amazon Soil Fosmids Plate#2, June 2013 Assem)	-2.5871	-49.041	3532881	3698	0	0.000
3300002157	Saline desert soil microbial communities from Kutch, Gujarat, India - S6	23.940972	70.188444	300833452	2224072	0	0.000
3300003065	Soil viral communities from Rice paddy at Daejon City	36.3875	127.3392	611313	1249	0	0.000
3300003102	Soil and Ice psychrophilic microbial communities from Leh Laddakh, India - psychrophilic sample	34.1453972	77.5676139	3997411	11352	0	0.000
3300005699	Soil microbial communities from Charlotte, North Carolina, that are pyrene degrading (re-annotation)	35.2269444	-80.8433333	5507702	9489	0	0.000
3300006427	Microbial communities of the formation fluids of a supercritical CO2 deposit from McElmo Dome, Colorado	39.7640021	-105.1353074	10773225	12085	0	0.000
3300006428	Microbial communities of the formation fluids of a supercritical CO2 deposit from Well 3, McElmo Dome, Colorado	39.7640021	-105.1353074	26625403	30536	0	0.000
	**Marine metagenomes**						
3300000555	Wetland microbial communities from Twitchell Island in the Sacramento Delta, sample from surface sediment Feb2011 Site A1 Bulk (Wetland Surface Sediment Feb2011 Site A1 Bulk-1% Merged Rds,0.27kb Insert)	38.107057	-121.647578	0	0	1	n.d
3300000573	Wetland microbial communities from Twitchell Island in the Sacramento Delta, sample from surface sediment Feb2011 Site A1 Bulk (Wetland Surface Sediment Feb2011 Site A1 Bulk-1% Merged Rds,0.25kb Insert)	38.107057	-121.647578	0	0	1	n.d
3300002359	Wetland sediment microbial communities	38.1072	-121.6485	0	0	44	n.d

	from Twitchell Island in the Sacramento Delta, sample from surface sediment Aug2011 Site B2 Bulk						
3300000444	Marine sediment microbial community from La Parguera, Puerto Rico - PR Tt Sediment 1 (PR Tt Sediment 1 - Bioluminescent Bay in La ParagueraPR, July 2012 Assem)	17.9675	-67.018833	10852089	26822	1	0.0921
3300001939	Marine microbial communities from Block Island, New York, USA - GS009	41.09111	-71.60222	37747991	70271	3	0.0795
3300006559	Marine microbial communities from the Black Sea in Odessa region - Od_3 (Assembly)	46.440968	30.772294	15417702	57359	1	0.0649
3300001935	Marine microbial communities from Northern Gulf of Maine, Canada - GS007	43.63222	-66.84722	32214377	54370	2	0.0621
3300000929	Marine plume microbial communities from the Columbia River - 15 PSU (Columbia River plume metagenome 15 PSU)	46.239	-124.161	448564426	1055341	26	0.0580
3300003517	Marine microbial communities from Antarctic Ocean - Station_363 -- 3.0 um (Cold Waters_1)	-60.00007	141.23353	86297686	253569	5	0.0579
3300001969	Marine microbial communities from Yucatan Channel, Mexico - GS017	20.5225	-85.41361	174719720	322314	10	0.0572
3300001964	Marine microbial communities from Rosario Bank, Honduras - GS018	18.036667	-83.78472	105952902	195732	6	0.0566
3300001957	Marine microbial communities from Wolf Island, Equador - GS035	1.3891667	-91.81695	88794277	150749	5	0.0563
3300000928	Marine plume microbial communities from the Columbia River - 25 PSU (Columbia River plume metagenome 25 PSU)	46.233	-124.16	465382374	999698	26	0.0559
3300000426	Marine sediment microbial community from Union City, CA, USA - Pond 1C Sediment 3 (Pond 1C Sediment 3 Union City, June 2012 Assem)	37.569083	-122.103267	90642744	224856	5	0.0552
3300001938	Marine microbial communities from Bedford Basin, Nova Scotia, Canada - GS005	44.690277	-63.637222	37780382	64231	2	0.0529
3300000101	Marine microbial communities from Delaware Coast, sample from Delaware MO Early Summer May 2010 (Delaware MO	39.0042816	-77.1012173	647909234	1375242	31	0.0478

	Early Summer May 2010, Feb 2012 assem)						
3300001952	Marine microbial communities from Newport Harbor, Rhode Island, USA - GS008	41.485832	-71.35111	85651166	142390	4	0.0467
3300001941	Marine microbial communities from Browns Bank, Gulf of Maine - GS003	42.85278	-66.217224	43542027	80361	2	0.0459
3300001961	Marine microbial communities from Dirty Rock, Cocos Island, Costa Rica - GS025	5.552778	-87.087776	88355364	163872	4	0.0453
3300001958	Marine microbial communities from Gulf of Mexico, USA - GS016	24.174723	-84.344444	88679928	152380	4	0.0451
3300001960	Marine microbial communities from South of Charleston, South Carolina, USA - GS014	32.506943	-79.263885	90953628	159679	4	0.0440
3300000115	Marine microbial communities from Delaware Coast, sample from Delaware MO Summer July 2011 (Delaware MO Summer July 2011, Nov 2011 assem)	38.848917	-75.1076	524209194	1204138	22	0.0420
3300000864	Marine plume microbial communities from the Columbia River - Metatranscriptome 25 PSU (Columbia River plume metatranscriptome 25 PSU)	46.235	-124.16	24148901	56425	1	0.0414
3300000883	Estuary microbial communities from the Columbia River - 5 PSU (Columbia River estuary metagenome 5 PSU)	46.235	-123.91	339832607	816259	14	0.0412
3300001943	Marine microbial communities from Cape May, New Jersey, USA - GS010	38.94	-74.685	57052860	104685	2	0.0351
3300000093	Wetland microbial communities from Twitchell Island in the Sacramento Delta, sample from surface sediment Feb2011 Site B2 Bulk (Wetland Surface Sediment Feb2011 Site B2 Bulk, Oct 2011 assem)	38.10726	-121.648538	61310069	187990	2	0.0326
3300000312	Wetland microbial communities from Twitchell Island in the Sacramento Delta, sample from surface sediment Feb2011 Site B2 Bulk (Wetland Surface Sediment Feb2011 Site B2 Bulk, Assem Ctgs Oct 2011 assem)	38.10726	-121.648538	61310069	187974	2	0.0326

3300000116	Marine microbial communities from Delaware Coast, sample from Delaware MO Spring March 2010 (Delaware MO Spring March 2010, Nov 2011 assem)	38.848917	-75.1076	590073671	1417215	19	0.0322
3300001945	Marine microbial communities from Galapagos, Equador - GS026	1.2641667	-90.295	63080825	112133	2	0.0317
3300000094	Wetland microbial communities from Twitchell Island in the Sacramento Delta, sample from surface sediment Feb2011 Site A1 Tule (Wetland Surface Sediment Feb2011 Site A1 Tule, Oct 2011 assem)	38.107057	-121.647578	129333279	362721	4	0.0309
3300000311	Wetland microbial communities from Twitchell Island in the Sacramento Delta, sample from surface sediment Feb2011 Site A1 Bulk (Wetland Surface Sediment Feb2011 Site A1 Bulk, Asm Ctgs IBYY,IIYG 2012 Mar Assem)	38.107057	-121.647578	243213055	583100	7	0.0288
3300000895	Wetland microbial communities from Twitchell Island in the Sacramento Delta, sample from surface sediment Feb2011 Site A1 Bulk (Wetland Surface Sediment Feb2011 Site A1 Bulk, Ctgs/Rds IBYY,IIYG 2012 Mar Assem)	38.107057	-121.647578	243213055	583095	7	0.0288
3300001949	Marine microbial communities from Panama City, Panama - GS022	6.492778	-82.90389	76322024	131400	2	0.0262
3300000310	Wetland microbial communities from Twitchell Island in the Sacramento Delta, sample from surface sediment Feb2011 Site A1 Bulk (Wetland Surface Sediment Feb2011 Site A1 Bulk, Assem Ctgs IIYG 2012 March Assem)	38.107057	-121.647578	120209763	331296	3	0.0250
3300000840	Wetland microbial communities from Twitchell Island in the Sacramento Delta, sample from surface sediment Feb2011 Site A1 Bulk (Wetland Surface Sediment Feb2011 Site A1 Bulk, Ctgs/UnRds IIYG 2012 March Assem)	38.107057	-121.647578	120209763	331295	3	0.0250

3300001940	Marine microbial communities from Bay of Fundy, Nova Scotia, Canada - GS006	45.111668	-64.94666	40280931	72308	1	0.0248
3300001956	Marine microbial communities from Rangirora Atoll, Polynesia Archipelagos - GS051	-15.143611	-147.435	83225611	150651	2	0.0240
3300000786	Wetland microbial communities from Twitchell Island in the Sacramento Delta, sample from surface sediment Feb2011 Site A2 Cattail (Wetland Surface Sediment Feb2011 Site A2 Cattail, Ctgs/UnReads Sept 2011 assem)	38.107057	-121.647578	84472288	260286	2	0.0237
3300001954	Marine microbial communities from Colon, Panama - GS019	10.716389	-80.25445	86398015	149187	2	0.0231
3300001962	Marine microbial communities from Cocos Island, Costa Rica - GS023	5.64	-86.56528	93108667	176476	2	0.0215
3300000117	Marine microbial communities from Delaware Coast, sample from Delaware MO Winter December 2010 (Delaware MO Winter December 2010, Nov 2011 assem)	39.0042816	-77.1012173	575155359	1341985	12	0.0209
3300000108	Wetland microbial communities from Twitchell Island in the Sacramento Delta, sample from surface sediment Feb2011 Site A1 Bulk (Wetland Surface Sediment Feb2011 Site A1 Bulk, 2011 Sep Assem)	38.107057	-121.647578	208340005	570282	4	0.0192
3300000309	Wetland microbial communities from Twitchell Island in the Sacramento Delta, sample from surface sediment Feb2011 Site A1 Bulk (Wetland Surface Sediment Feb2011 Site A1 Bulk, Assem Ctgs IBYY 2011 Sep Assem)	38.107057	-121.647578	208340005	570282	4	0.0192
3300000854	Wetland microbial communities from Twitchell Island in the Sacramento Delta, sample from surface sediment Feb2011 Site B2 Tule (Wetland Surface Sediment Feb2011 Site B2 Tule, Ctgs/UnReads Oct 2011 assem)	38.10726	-121.648538	105539119	321832	2	0.0190

3300002053	Marine sediment microbial communities from White Oak River estuary, North Carolina - WOR_SMTZ	34.6478111	-77.1112083	799007063	857807	12	0.0150
3300000894	Wetland microbial communities from Twitchell Island in the Sacramento Delta, sample from surface sediment Feb2011 Site L1 Bulk (Wetland Surface Sediment Feb2011 Site L1 Bulk, Ctgs/UnReads Jan 2012 assem)	38.106796	-121.646457	70007605	199148	1	0.0143
3300002961	Wetland microbial communities from Twitchell Island in the Sacramento Delta, sample from surface sediment Feb2011 Site A1 Bulk (Wetland microbial communities from Twitchell Island in the Sacramento Delta, sample from surface sediment Feb2011 Site A1 Bulk, ASSEMBLY_DATE=20140701)	38.107057	-121.647578	560427570	1398510	8	0.0143
3300000917	Wetland microbial communities from Twitchell Island in the Sacramento Delta, sample from surface sediment Feb2011 Site A2 Cattail (Wetland Surface Sediment Feb2011 Site A2 Cattail, Ctgs/UnRds Jan 2012 Assem)	38.107057	-121.647578	146925476	408994	2	0.0136
3300003516	Marine microbial communities from Antarctic ocean - Station_363 -- 0.8 um (Cold Waters_2)	-60.00007	141.23353	77909508	239389	1	0.0128
3300000077	Wetland microbial communities from Twitchell Island in the Sacramento Delta, sample from surface sediment Feb2011 Site B1 Bulk (Wetland Surface Sediment Feb2011 Site B1 Bulk Feb 2012)	38.10726	-121.648538	79467785	197107	1	0.0126
3300001968	Marine microbial communities from Lake Gatun, Panama - GS020	9.164444	-79.83611	166403274	296358	2	0.0120
3300001955	Marine microbial communities from Gulf of Panama, Panama - GS021	8.129167	-79.69111	85347158	150154	1	0.0117

3300003432	Wetland sediment microbial communities from Twitchell Island in the Sacramento Delta, sample from surface sediment Aug2011 Site B2 Bulk (Wetland sediment microbial communities from Twitchell Island in the Sacramento Delta, sample from surface sediment Aug2011 Site B2 Bulk, ASSEMBLY_DATE=20140909)	38.1072	-121.6485	2212415794	5001746	23	0.0104
3300003541	Wetland sediment microbial communities from Twitchell Island in the Sacramento Delta, sample from surface sediment Aug2011 Site B2 Bulk (Wetland sediment microbial communities from Twitchell Island in the Sacramento Delta, sample from surface sediment Aug2011 Site B2 Bulk, ASSEMBLY_DATE=20141008)	38.1072	-121.6485	1842474917	3667351	18	0.0098
3300000092	Wetland microbial communities from Twitchell Island in the Sacramento Delta, sample from surface sediment Feb2011 Site L2 Tule (Wetland Surface Sediment Feb2011 Site L2 Tule, Sep 2011 assem)	38.106796	-121.646457	111737537	334657	1	0.0089
3300000317	Wetland microbial communities from Twitchell Island in the Sacramento Delta, sample from surface sediment Feb2011 Site L2 Tule (Wetland Surface Sediment Feb2011 Site L2 Tule, Assem Ctgs Sep 2011 assem)	38.106796	-121.646457	111737537	334653	1	0.0089
3300000425	Marine microbial community from Union City, CA, USA - Pond 2C Liquid 2 (Pond 2C Liquid 2 Union City, July 2012 Assem)	37.569017	-122.102433	131906500	297522	1	0.0076
3300001965	Marine microbial communities from Coastal Floreana, Equador - GS028	-1.2169445	-90.319725	135719211	247934	1	0.0074
3300000090	Wetland microbial communities from Twitchell Island in the Sacramento Delta, sample from surface sediment Feb2011 Site B1 Cattail (Wetland Surface Sediment Feb2011 Site B1 Cattail, Sep 2011 assem)	38.10726	-121.648538	291270262	747850	2	0.0069

3300000313	Wetland microbial communities from Twitchell Island in the Sacramento Delta, sample from surface sediment Feb2011 Site B1 Cattail (Wetland Surface Sediment Feb2011 Site B1 Cattail, Assem Ctgs Sep 2011 assem)	38.10726	-121.648538	291270262	747866	2	0.0069
3300000030	Wetland microbial communities from Twitchell Island in the Sacramento Delta, sample from surface sediment Feb2011 Site B1 Bulk (Wetland Surface Sediment Feb2011 Site B1 Bulk, Oct 2011 assem)	38.10726	-121.648538	153197283	433712	1	0.0065
3300001281	Wetland microbial communities from Twitchell Island in the Sacramento Delta, sample from surface sediment Aug2011 Site A1 Bulk (Wetland Surface Sediment Aug2011 Site A1 Bulk Metagenome, ASSEMBLY_DATE=20130408)	38.107	-121.6475	173204638	479751	1	0.0058
3300000418	Marine microbial community from Union City, CA, USA - Pond 2C Liquid 1 (Pond 2C Liquid 1 Union City, June 2012 Assem)	37.569167	-122.1019	194495031	424469	1	0.0051
3300000385	Marine microbial community from Cabo Rojo, Puerto Rico - PR CR 10% Liquid 1 (PR CR 10% Liquid 1 Cabo Rojo PR, June 2012 Assem)	17.951083	-67.193167	210351197	409868	1	0.0048
3300003475	Marine microbial communities from the Indian Ocean - GS112 (warm waters)	-8.505	80.37556	224938505	730224	1	0.0044
3300000091	Wetland microbial communities from Twitchell Island in the Sacramento Delta, sample from surface sediment Feb2011 Site L1 Cattail (Wetland Surface Sediment Feb2011 Site L1 Cattail, Sep 2011 assem)	38.106796	-121.646457	296519018	679298	1	0.0034
3300000318	Wetland microbial communities from Twitchell Island in the Sacramento Delta, sample from surface sediment Feb2011 Site L1 Cattail (Wetland Surface Sediment Feb2011 Site L1 Cattail, Assem Ctgs Sep 2011 assem)	38.106796	-121.646457	296519018	679305	1	0.0034

2199352009	Marine subseafloor sediment microbial communities, sample from White Oak River Estuary, NC, USA 14E (White Oak River Estuary June 2011 assem)	34.690251	-77.106571	22398301	51820	0	0.0000
3300000100	Wetland microbial communities from Twitchell Island in the Sacramento Delta, sample from surface Sediment Feb2011 Site B2 Cattail (Wetland Surface Sediment Feb2011 Site B2 Cattail, Oct 2011 assem)	38.10726	-121.648538	90589564	284067	0	0.0000
3300000316	Wetland microbial communities from Twitchell Island in the Sacramento Delta, sample from surface Sediment Feb2011 Site B2 Cattail (Wetland Surface Sediment Feb2011 Site B2 Cattail, Assem Ctgs Oct 2011 assem)	38.10726	-121.648538	90589564	284068	0	0.0000
3300000320	Marine microbial communities from Delaware Coast, sample from Delaware MO Early Summer May 2010	39.0042816	-77.1012173	3880822	10893	0	0.0000
3300000321	Marine microbial communities from Delaware Coast, sample from Delaware MO Winter December 2010	39.0042816	-77.1012173	11256121	45129	0	0.0000
3300000369	Marine microbial community from Union City, CA, USA - Pond 2C Liquid 3 (Pond 2C Liquid 3 Union City, June 2012 Assem)	37.568817	-122.10315	17102308	18170	0	0.0000
3300000371	Marine microbial community from Union City, CA, USA - Pond 1C Liquid 3 (Pond 1C Liquid 3 Union City, June 2012 Assem)	37.5693	-122.102517	47212914	125124	0	0.0000
3300000403	Marine sediment microbial community from La Parguera, Puerto Rico - PR Tt Sediment 2 (PR Tt Sediment 2 - Bioluminescent Bay in La ParagueraPR, July 2012 Assem)	17.967317	-67.018833	898328	2770	0	0.0000
3300000409	Marine sediment microbial community from Union City, CA, USA - Pond 2C Sediment 1 (Pond 2C Sediment 1 Union City, June 2012 Assem)	37.569167	-122.1019	24779847	77851	0	0.0000
3300000463	Marine sediment microbial community from Union City, CA, USA - Pond 2C Sediment 3	37.568817	-122.10315	38427200	111900	0	0.0000

	(Pond 2C Sediment 3 Union City, June 2012 Assem)						
3300000517	Marine microbial community from Cabo Rojo, Puerto Rico - PR CR 10% Liquid 3 (PR CR 10% Liquid 3 Cabo Rojo PR, June 2012 Assem)	17.950617	-67.193417	120470807	242233	0	0.0000
3300000853	Marine plume microbial communities from the Columbia River - Metatranscriptome 15 PSU (Columbia River plume metatranscriptome 15 PSU)	46.239	-124.161	7327946	18177	0	0.0000
3300000867	Estuary microbial communities from the Columbia River - metatranscriptome 5 PSU (Columbia River estuary metatranscriptome 5 PSU)	46.235	-123.91	58151542	137277	0	0.0000
3300001279	Wetland sediment microbial communities from Twitchell Island in the Sacramento Delta, sample from surface sediment Aug2011 Site B2 Bulk (Wetland Surface Sediment Aug2011 Site B2 Bulk Metagenome, ASSEMBLY_DATE=20130408)	38.1072	-121.6485	217817654	615872	0	0.0000
3300001922	Marine microbial communities from Polynesia - GS046	-9.571111	-131.49167	336890	629	0	0.0000
3300001923	Marine microbial communities from the Tropical South Pacific Ocean - GS038	-2.5819445	-97.85139	451181	795	0	0.0000
3300001924	Marine microbial communities from Tikehau Lagoon, Polynesia Archipelagos - GS050	-15.277778	-148.22444	467458	887	0	0.0000
3300001925	Marine microbial communities from the Tropical South Pacific Ocean - GS041	-5.93	-108.68694	494549	909	0	0.0000
3300001926	Marine microbial communities from the Tropical South Pacific Ocean - GS042	-7.1075	-116.11916	542583	953	0	0.0000
3300001927	Marine microbial communities from Polynesia - GS044	-8.415	-124.23972	562395	1015	0	0.0000
3300001928	Marine microbial communities from the Tropical South Pacific Ocean - GS043	-7.661111	-116.11916	612652	1073	0	0.0000
3300001930	Marine microbial communities from the Tropical South Pacific Ocean - GS039	-3.3433332	-101.373886	641644	1142	0	0.0000

3300001931	Marine microbial communities from Polynesia - GS045	-9.0175	-127.76722	636185	1152	0	0.0000
3300001932	Marine microbial communities from Moorea, Cooks Bay, Polynesia Archipelagos - GS049	-17.453056	-149.79889	638416	1194	0	0.0000
3300001933	Marine microbial communities from Moorea, Cooks Bay - GS048a	-17.475834	-149.81223	666592	1245	0	0.0000
3300001936	Marine microbial communities from Halifax, Nova Scotia, Canada - GS004	44.137222	-63.644444	33043040	56286	0	0.0000
3300001937	Marine microbial communities from the Equatorial Pacific Ocean - GS037	-1.9738889	-95.014725	36191958	63262	0	0.0000
3300001947	Marine microbial communities from the Gulf of Maine, Canada - GS002	42.503056	-67.24	74202974	125810	0	0.0000
3300001950	Marine microbial communities from Delaware Bay, New Jersey, USA - GS011	39.417778	-75.504166	79799345	138086	0	0.0000
3300001953	Marine microbial communities from Key West, Florida, USA - GS015	24.488333	-83.07	84693422	146422	0	0.0000
3300001959	Mangrove swamp microbial communities from Isabella Island, Equador - GS032	-0.5938889	-91.06944	89398486	153552	0	0.0000
3300003066	Marine Synechococcus microbial communities from coastal surface water at La Jolla, California, USA - SRS00536	32.850317	-117.27494	307378	1337	0	0.0000
3300003073	Marine surface microbial communities Puget Sound, Washington, USA	47.6906558	-122.404411	2059964	1892	0	0.0000
3300003098	Marine microbial communities from surface seawater at Gulf of Maine	43.14	-68.33	5278992	8597	0	0.0000
3300003139	Mat microbial community in the pink berry consortia of the Sippewissett salt marsh	41.575836	-70.63923	33200272	56503	0	0.0000
3300004831	Marine surface microbial communities from the North Atlantic Ocean - filtered matter	41.226956	-8.720528	27429400	84353	0	0.0000
3300005472	Wetland microbial communities from Twitchell Island in the Sacramento Delta, sample from surface sediment Feb2011 Site L1 Cattail (re-annotation)	38.106796	-121.646457	2364101	2630	0	0.0000
3300005698	Marine microbial communities from six Antarctic regions - DNA Fragments (re-annotation)	-64.766667	-64.05	43087	39	0	0.0000

3300005722	Marine Trichodesmium cyanobacterial communities from the North Pacific Subtropical Gyre outside Oahu, HI, sample from new species B colonies (re-annotation)	22.75	-158	27189071	39432	0	0.0000
3300006561	Marine microbial communities from the Black Sea in Odessa region - Od_1 (Assembly)	46.35116	30.5519	22413229	75560	0	0.0000
3300006562	Marine microbial communities from the Black Sea in Odessa region - Od_2 (Assembly)	46.302094	30.668915	31351919	105939	0	0.0000
